# Comparative Analysis of Beamforming Techniques and Beam Management in 5G Communication Systems

**DOI:** 10.3390/s25154619

**Published:** 2025-07-25

**Authors:** Cristina Maria Andras, Gordana Barb, Marius Otesteanu

**Affiliations:** Department of Communications, Politehnica University of Timisoara, 300006 Timisoara, Romania; cristina.andras@upt.ro (C.M.A.); marius.otesteanu@upt.ro (M.O.)

**Keywords:** beamforming, beam management, beam determination, beam sweeping, spectral spectrogram, synchronization signal blocks

## Abstract

The advance of 5G technology marks a significant evolution in wireless communications, characterized by ultra-high data rates, low latency, and massive connectivity across varied areas. A fundamental enabler of these capabilities is represented by beamforming, an advanced signal processing technique that focuses radio energy to a specific user equipment (UE), thereby enhancing signal quality—crucial for maximizing spectral efficiency. The work presents a classification of beamforming techniques, categorized according to the implementation within 5G New Radio (NR) architectures. Furthermore, the paper investigates beam management (BM) procedures, which are essential Layer 1 and Layer 2 mechanisms responsible for the dynamic configuration, monitoring, and maintenance of optimal beam pair links between gNodeBs and UEs. The article emphasizes the spectral spectrogram of Synchronization Signal Blocks (SSBs) generated under various deployment scenarios, illustrating how parameters such as subcarrier spacing (SCS), frequency band, and the number of SSBs influence the spectral occupancy and synchronization performance. These insights provide a technical foundation for optimizing initial access and beam tracking in high-frequency 5G deployments, particularly within Frequency Range (FR2). Additionally, the versatility of 5G’s time-frequency structure is demonstrated by the spectrogram analysis of SSBs in a variety of deployment scenarios. These results provide insight into how different configurations affect the synchronization signals’ temporal and spectral occupancy, which directly affects initial access, cell identification, and energy efficiency.

## 1. Introduction

In recent years, radio communication systems have experienced a significant increase in the number of users along with the demand for new applications, service types such as enhanced mobile broadband (eMBB), ultra-reliable low-latency communication (URLLC), and massive machine type communication (mMTC), as well as advanced data services. To optimize throughput in modern communication systems, it is essential to maximize both bandwidth and spectrum efficiency. As spectrum becomes increasingly crowded and the demand for higher data rates grows, enhancing spectrum efficiency has become a critical goal [1]. One widely adopted strategy to achieve this is the deployment of multiple antennas at both the transmitter and receiver side, commonly known as Multiple-Input Multiple-Output (MIMO) technology.

The current mobile generation, 5G, represents a significant evolution in wireless systems, with vast improvements over previous generations such as 4G and 3G. A key advantage of 5G is the use of the Millimeter-Wave (mmWave) spectrum, with frequencies ranging from 30 to 300 GHz, which provides a much larger bandwidth compared to traditional sub-6 GHz frequencies [2]. Moreover, it addresses the primary issue of the sub-6 GHz spectrum being already congested. This way, higher data transfer rates are achieved, and more efficient communication is enabled [3]. On the downside, mmWave frequencies suffer from significant propagation losses, particularly in outdoor environments. To mitigate this challenge, advanced technologies such as massive MIMO and beamforming are implemented, improving signal strength and enhancing the overall performance of the mmWave spectrum [4].

Massive MIMO is defined by implementing a large number of antennas, usually dozens or even hundreds, at the base station (gNodeB) to simultaneously serve multiple users through spatial multiplexing [5]. This not only improves the system’s capacity but also provides better signal quality by minimizing interference and optimizing the usage of available spectrum. The key benefit of massive MIMO lies in its ability to have independent communication channels for each user in the same frequency band, which significantly increases data throughput [6]. Additionally, the vast number of antennas enables spatial filtering, which helps in reducing the impact of signal fading and improving coverage, especially in environments with challenging propagation conditions.

Beamforming, on the other hand, is a signal processing technique that directs the transmission and reception of the signal in a specific desired direction, rather than sending the signal uniformly in all directions. This way, the radiated antenna’s beam patterns are dynamically focused on the intended user equipment while minimizing interference from unwanted signals [7]. This is accomplished by assigning specific weights to individual antenna elements, typically calculated using digital signal processing algorithms that estimate the direction of arrival (DoA) of incoming signals. By identifying the optimal transmission path, beamforming minimizes interference caused by closely spaced transmitters and receivers. A key enabler of this capability is beam management, which plays a critical role in simplifying initial access and maintaining robust connectivity by periodically identifying the optimal beam pair between the UE and the gNodeB. Additionally, the effectiveness of beamforming also depends on the method used to compute and apply the weights. Adaptive beamforming techniques adjust in real time to changing signal conditions, optimizing spatial response and communication quality. On the other hand, non-adaptive techniques use fixed parameters and are less effective at mitigating interference or maintaining high performance [8,9]. The combined use of massive MIMO and beamforming technologies is expected to be an essential part of future wireless communication systems, ensuring high performance, low latency, and reliable connectivity. Furthermore, these technologies contribute to the efficient use of frequency spectrum and the management of high-density networks, which are crucial to meeting the eMBB, URLLC, and mMTC services in 5G and beyond. Standardization efforts, such as those led by 3GPP, play a critical role in ensuring interoperability and seamless integration of these technologies into the global network infrastructure [10].

The structure of the paper is organized as follows: Section 2 introduces and classifies beamforming techniques relevant to 5G systems. Section 3 discusses beam management procedures, detailing key operations such as beam sweeping, measurement, determination, reporting, and recovery. Section 4 presents simulation results, including spectrogram analysis of synchronization signal blocks (SSBs), 3D directivity patterns, and reference signal received power (RSRP) evaluations across multiple scenarios. Finally, Section 5 concludes the study by summarizing key findings and highlighting the impact of advanced beamforming and beam management strategies on 5G system performance.

## 2. Beamforming Techniques and Classification

**Figure 1:**
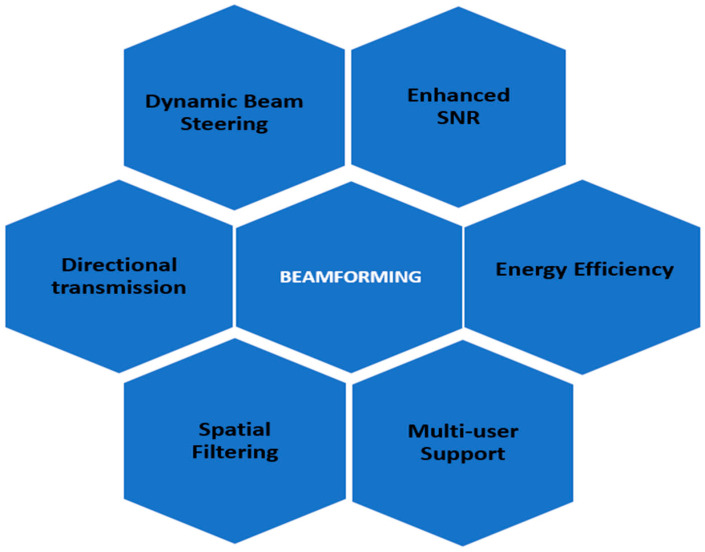
Beamforming: enhancing wireless efficiency and performance.

Beamforming is a fundamental technique in modern wireless communication systems that enables focused and efficient signal transmission. As shown in Figure 1, beamforming enhances system performance by offering directional transmission, improved signal-to-noise ratio (SNR), and energy efficiency. Moreover, it also supports dynamic beam steering, spatial filtering, and multi-user communication, making it well-suited for dense network environments and next-generation technologies such as 5G and beyond [11]. Dynamic beam steering refers to the real-time adjustment of the beam’s direction based on the receiver’s location, allowing the system to maintain a strong and focused link even when users move [12].

This adaptability significantly improves coverage and link reliability in mobile environments. Spatial filtering enables the system to selectively receive or transmit signals from specific spatial directions while suppressing interference from others. Isolating desired signals and mitigating noise enhances signal quality and spectral efficiency. Multi-user support allows the system to serve multiple users simultaneously by forming separate beams for each user, reducing interference and increasing the network’s overall capacity. Directional transmission ensures that energy is concentrated in the intended direction of communication rather than being broadcast omnidirectionally, resulting in improved energy efficiency and reduced interference with other users [13].

Beamforming techniques can be classified based on various criteria, including the signal domain, location, bandwidth, channel estimation, and the method of applying weight vectors, as summarized in Table 1.

The demand for quicker, more reliable connections is increasing. Beamforming, a signal processing technology utilized in modern wireless systems, has emerged as an important answer for meeting this requirement. This introduction investigates the concepts of beamforming and its transformative role in improving the performance of technologies such as Wi-Fi, 5G, and more, as presented in Figure 1.

Table 2 offers an in-depth view of each of the above-mentioned beamforming techniques and a description of the main characteristics.

**Figure 2:**
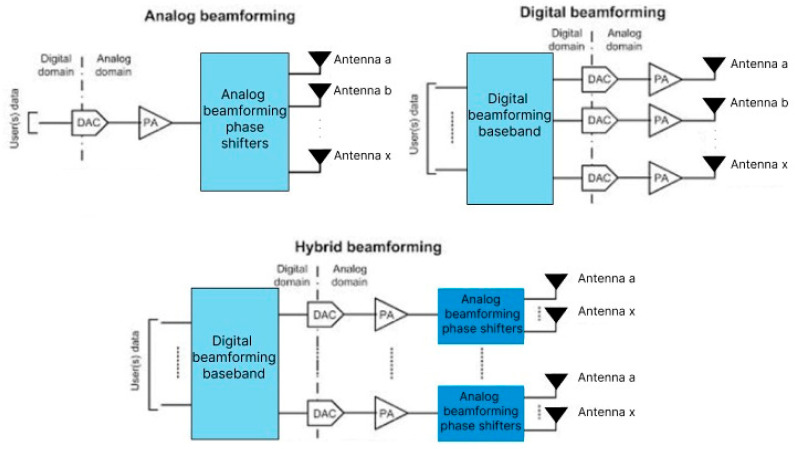
Beamforming architectures [22].

Due to various propagation characteristics, including significant free space path loss, absorption by atmospheric gases and rain, and non-line of sight (NLOS) propagation, beamforming has become essential in the above 6 GHz frequency bands, even though it can also be employed in the sub-6 GHz frequency bands alongside massive MIMO. Beamforming can be implemented at either the base station or the user equipment, as already discussed, depending on the application. By dynamically adjusting the phase shifts in the transmitted and received signals, both the transmitter and receiver can more accurately steer the beam toward a desired direction [20]. This enables enhanced beam gain toward the intended UEs while suppressing interference from redundant spatial directions. This approach can be realized using analog, digital, or hybrid beamforming architectures, as illustrated in Figure 2.

The analog architecture comprises a phased array with only one radio frequency (RF) chain, controlled by digital-to-analog converters (DAC) in the case of the transmitter or analog-to-digital converters (ADC) when it comes to the receiver. The data converters and RF chain handle only one stream of input data, which is then distributed on multiple paths, according to the number of antenna elements. Each of these signals then goes through one of the phase shifters before being amplified and transmitted by the array system. Analog beamforming is an elementary and cost-effective method to guarantee accurate data transmission, owing to the small amount of hardware equipment needed. Because of these weaknesses, analog beamforming is considered suitable for indoor or point-to-point solutions. In fully analog beamforming architectures, power amplifiers (PAs) and digital phase shifters (PSs) are used to reduce system complexity and power consumption [21]. Adaptive beamforming can be applied at either the BS (Base Station) or UE to establish the optimal beam pair for connectivity. By adjusting the phase of transmitted and received signals, TX and RX can better control the direction of constructive interference—maximizing gain toward target UEs while minimizing interference from unwanted directions. This is implemented using analog, digital, or hybrid architecture, as presented in Figure 2.

Digital beamforming techniques, which are based on sophisticated signal processing algorithms, are used when several cochannel signals reach an antenna array. It reduces array gain in the direction of the Signals Not of Interest (SNoI) and increases array directivity in the direction of the Signal of Interest (SoI). Either supervised (non-blind) or unsupervised (blind) learning techniques are used to estimate the beamforming weights. Digital beamforming stands out as the most robust and capable architecture in terms of flexibility, as it supports multiple simultaneous data streams and offers the ability to implement advanced signal processing techniques. In this case, there are multiple RF chains, each of them linked to a DAC or an ADC and a phase shifter. Despite these advantages, digital beamforming often incurs higher power consumption and increased hardware costs due to the complexity of antenna arrays and signal processing requirements [23].

Hybrid beamforming (HB) divides signal processing tasks between the digital and analog domains, effectively balancing flexibility and efficiency [24]. Hybrid beamforming improves channel gain while minimizing interference at a tolerable complexity. HB in Massive MIMO (mMIMO) systems aims to maximize the sum rate that can approach the fully digital beamforming system performance. Combining mMIMO systems, mmWave, and HB methods improves data speeds and cell coverage in 5G wireless networks. This reduces hardware complexity, energy consumption, and costs by reducing the number of radio-frequency chains needed at the base station [25]. By leveraging the strengths of both approaches, hybrid architecture reduces hardware complexity, particularly valuable in massive MIMO systems—and is thus considered a promising solution. In this framework, each data stream is assigned a dedicated analog beamforming unit (BFU) connected to a subset of antennas, enabling energy-efficient operation while minimizing the number of required RF chains and maintaining system performance [26].

Furthermore, the path to 6G wireless networks requires solving major issues in spectrum efficiency, network densification, energy consumption, and signal dependability in complex propagation settings. Recent studies show that reconfigurable Intelligent Surfaces (RISs) and AI-driven beamforming are critical components of next-generation wireless networks, particularly for 6G integration. These technologies improve communication efficiency in challenging environments by allowing for dynamic signal transmission regulation. As an example of how beamforming techniques are evolving to meet demanding real-time and precision requirements in high-mobility scenarios, the work in [27] demonstrates harmonic coordinated beamforming for time-modulated arrays in automotive radar, which improves dual-lane detection capabilities.

Similarly, Reconfigurable Intelligent Surfaces are programmable meta-surfaces that can reflect incoming electromagnetic waves with variable phase shifts. RIS technology provides significant advantages, such as improving non-line-of-sight (NLoS) connectivity and optimizing the wireless environment to reduce path loss [28]. In [29], self-powered absorptive RISs are proposed to increase security in satellite–terrestrial integrated networks (STINs), which are a critical architecture for 6G. RIS are designed to operate independently, signifying a move toward sustainable and intelligent electromagnetic environments. Low-power reconfiguration helps to support green communication. Self-powered devices, meta-material-based control, and RIS-user scheduling are all examples of cutting-edge research in RIS. These surfaces are integrated into terrestrial, UAV-assisted, and satellite networks, providing strong signal connectivity and improved physical-layer security.

To facilitate adaptive, real-time decision-making in complex and dynamic situations, beamforming systems are increasingly incorporating AI and machine learning. In contrast to conventional algorithms, AI-based beamforming is capable of assuming mobility conditions and forecasting the best beam directions; the CSI acquisition overhead should be decreased, particularly in large MIMO systems [30].

An interesting research topic is the integration of AI and RIS, which will result in intelligent environments in which the RIS setup adapts in real time using AI models. These systems offer potential in smart radio environment orchestration, low-latency URLLC applications, and cooperative beam management using UAVs or satellites. These studies emphasize the growing importance of intelligent, adaptive radio environments in future networks and advocate for their inclusion in any analysis of MIMO and massive MIMO systems in 6G scenarios [31].

## 3. SSB Set and Beam Selection

The paper’s literature review was divided into several areas, including channel state estimation, beam sweeping, beam determination, beam reporting, and beam recovery. The thorough completion of the procedures above mentioned results in beam management, which establishes a robust communication channel between the base station and the user equipment. Since it enables signal quality optimization, channel state estimation is an essential part of any telecommunications system. As the number of antenna arrays increases, channel estimation becomes more challenging. Auxiliary Beam Pair (ABP), few-bit ADC architecture, adaptive algorithm-based codebook design, least-square estimators, maximum probability estimators, and a multi-resolution codebook were the conventional approaches used for channel estimation. A range of compressed sensing techniques can also be used for channel estimation. To provide an increased resolution of angles, ABP is employed [32].

System timing and radio frame synchronization are essential steps when a UE attempts to access a cell for the first time. This process begins with the detection of the primary synchronization signal (PSS), which comprises one of three possible sequences. These sequences are generated by applying three different cyclic shifts to a base sequence of 127 BPSK-modulated symbols, mapped onto 127 Resource Elements (REs).

Successfully detecting the PSS enables the UE to determine the transmission timing of the secondary synchronization signal (SSS), which corresponds to one of 336 possible sequences. Combined, the PSS and SSS facilitate the identification of the unique physical cell identity (PCI) of the detected cell [33]. The PCI serves as a physical layer identifier within the 5G radio access network (RAN) and is critical for downlink synchronization and channel estimation [34]. Ultimately, one of the 1008 possible cell identity values can be computed based on the detected PSS and SSS indices [35]. In 5G systems, PCI is calculated using the indices of the PSS and the SSS as follows:

*N*_*Cell*_*ID*_ = 3 × *N*_1_*ID*_ + *N*_2___*ID*_ (1)

where

- *N*_1___*ID*_ relates to the SSS with the range {0, 1….335};
- *N*_2___*ID*_ relates to the PSS with the range {0, 1, 2}.

A synchronization signal block is periodically transmitted—typically every 20 ms by defaulting over a specific set of time and frequency REs within the basic OFDM grid. It occupies four consecutive OFDM symbols in the time domain and 240 contiguous subcarriers (equivalent to 20 resource blocks, each with 12 subcarriers) in the frequency domain. In case of bands with two possible subcarrier spacings (SCSs), the UE must attempt to use both to detect the SSB. This 20 ms default value is used as an indication for the UE to know how long it must stay on each frequency before concluding that there are no synchronization signals (SSs) present. However, the periodicity of SSB transmissions can consist of shorter or longer intervals, ranging from 5 ms to 160 ms. This flexibility is favorable in various scenarios—for instance, enabling faster cell search procedures in devices already connected to the network, where beam sweeping involves multiple beams and many time-multiplexed SS blocks. Alternatively, longer periodicities can enhance energy efficiency in mMTC use cases [36].

**Figure 3:**
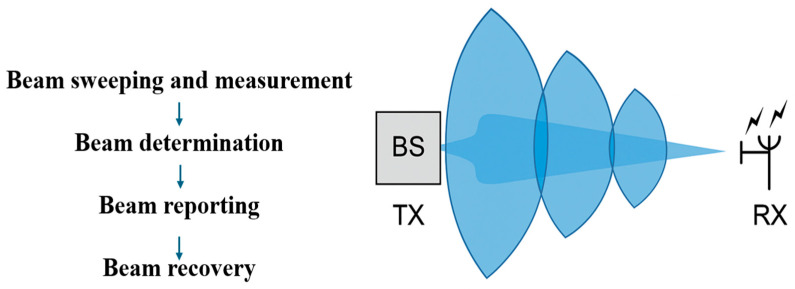
Fifth-generation beam management procedure.

Beam management means a series of procedures at Layer 1 and 2 that are applied to establish and maintain a set of connections between two beams: one utilized at the gNodeB and one used at the UE [37]. The included operations are beam sweeping, beam measurement, beam determination, beam reporting, and beam recovery, as presented in Figure 3. These procedures primarily address a range of control tasks, including identifying an appropriate beamformed beam-pair for idle users, connecting the device to the network, maintaining optimal connectivity for users who are already connected through beam tracking processes, or even beam recovery procedures that are required to quickly restore the connection following blockage events, gradual changes in the environment’s radio propagation conditions. As noted in [38], typical beam failure recovery times can range from 50 to 100 ms. This delay becomes problematic, especially in the use case of URLLC, which often requires end-to-end latencies well below 1 ms for mission-critical applications. Recent studies suggest that while URLLC-optimized configurations may reduce this to 10–40 ms, other device types (e.g., NB-IoT) still experience delays up to 200 ms. These latencies highlight the beam recovery mechanism’s limitations in highly dynamic environments such as urban canyons, where mobility and blockage further degrade responsiveness.

On the other hand, beam management in 5G NR, particularly at mmWave frequencies, requires significant signaling overhead due to the necessity for frequent beam measurements, reporting, and alignment. Specifically, uplink-based beam reporting systems can cost up to 15% of system resources, particularly when dealing with dense antenna arrays and huge beam codebooks. This overhead is triggered by the periodic transmission of Channel State Information–Reference Signals and sounding reference signals (SRS) based beam reporting, which are required to sustain resilient links under high mobility or frequent blockage. It is underlined how this overhead can affect system throughput and latency, making beam management efficiency an important design consideration for both 5G and future 6G systems [39].

Among the BM procedures defined by 3GPP, the first—initial beam establishment (IBE)—emphasizes the determination of the most efficient beam pair for both downlink and uplink communication [40].

The UE performs beam sweeping to determine and select the optimal beam during the initial access phase. A gNodeB emits a series of beams in all directions at consistent time intervals. This procedure addresses the initial beam acquisition for UE in idle mode, utilizing SSBs. During this phase, beam sweeping is performed at both the transmitter and receiver to identify the most suitable beam pair based on RSRP measurements. The UE acquires and interprets the SSB during network synchronization to extract PSS, SSS, physical broadcast channel (PBCH), and demodulation reference signal (DMRS).

After establishing the connection, these initial beams are further refined using CSI-RS for downlink and SRS for uplink. CSI refers to known channel parameters of radio links and describes scattering, diffraction, fading, shadowing, and other factors that occur when signals are propagated from a transmitter to its corresponding receiver over the air. CSI is utilized to assess the quality of a radio link.

To determine the best possible connection, beam-pair comparisons using pertinent metrics are essential in 5G systems. This highlights the importance of effective beam management, with UE and gNodeB identifying the best beams to operate on at any given time. Among the MB processes, a synchronization signal burst must first be generated. Each SSB within the burst is then beamformed to scan across both azimuth and elevation directions. Further, this beamformed signal is transmitted through a spatial scattering channel. On the receiver side, the signal is processed across multiple receiving beams. By measuring the RSRP for each transmit–receive beam pair, it is possible to identify the beam pair that yields the highest RSRP. This optimal beam pair represents the best link between the transmitter and receiver under the given spatial simulation scenario [39].

For the beam measurement and determination, the UE measures the received signal power, which determines the beam intensity. The UE tracks the ideal beam using specified criteria set by the gNodeB, identifying the beam with the highest RSRP. The UE reports the ideal beam to the gNodeB, a process known as beam reporting. A physical random-access channel (PRACH) is an uplink channel used for initial access or synchronization of disconnected mobile devices with the network. The UE broadcasts the PRACH preamble in inactive mode after selecting the beam for one or more PRACH intervals with a time and frequency offset. The UE sends the PRACH preamble tailored to the SS block with the best beam.

The UE is configured by the network to perform beam reporting at predefined intervals. This process enables the network to monitor link quality and ensure reliable connectivity. In scenarios where beam failure occurs—typically due to channel impairments such as blockage or fading—a beam recovery procedure is triggered. This mechanism aims to reestablish communication by selecting a new, viable beam pair. Upon detecting predefined failure conditions, the UE begins monitoring reference signals, such as CSI-RS or SSBs, to confirm the beam failure event and to assist in the selection of a new beam. Beam recovery is then performed, which involves dynamically adjusting the beam’s orientation or direction. This ensures that the communication link is maintained or quickly restored, thereby preserving service quality and minimizing latency [41].

**Figure 4:**
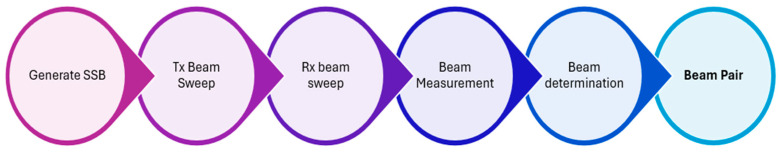
Simulated architecture of TX/RX beam management.

Figure 4 highlights, as a summary, the key signal processing stages involved in the dual-end beam sweeping procedure at both the gNodeB and UE.

In conclusion, the beam selection problem involves identifying the most suitable set of beams for communication between the transmitter and receiver, as well as the optimal antenna layout for a given situation. Another possible option will be the use of predetermined codebooks on both the transmitter and receiver sides. The codewords that result in the greatest gain for the current channel between the transmitter and receiver should be chosen from these codebooks. There is a time limit for sending control messages via the downlink of 5G New Radio. Training sequences are delivered to each beam at this time, and the mobile station determines which beam should be used for communication between them based on the power received; if the receiver also employs beamforming, it must select the optimal beam, so the process becomes even more complex [42,43].

## 4. Simulations and Results

This section provides the simulation setup adopted as well as the results obtained. To perform the simulations for this work, the Phased Array System Toolbox from Matlab2024b is used. To conduct the simulations presented in this study, the Phased Array System Toolbox and the 5G Toolbox provided by MATLAB2024b were employed.

The 5G spectrum is divided into two frequency ranges: frequency range (FR1) and frequency range (FR2) [44]. The initial range spans between 450 MHz and 6000 MHz. The second range, from 24.25 GHz to 52.6 GHz, has a modest wavelength of millimeters, making FR2 suitable for mmWaves. The 3GPP offers technical specifications for FR1 and FR2, which may be found in TS 38.101-1 [42] and TS 38.101-2 [45]. In frequency range 1, the maximum number is either 4 or 6 blocks per burst, while in frequency range 2, it can go up to 64 blocks per burst. These cases incorporate parameters that define each operating frequency, master information block (MIB), and the PBCH.

**Figure 5:**
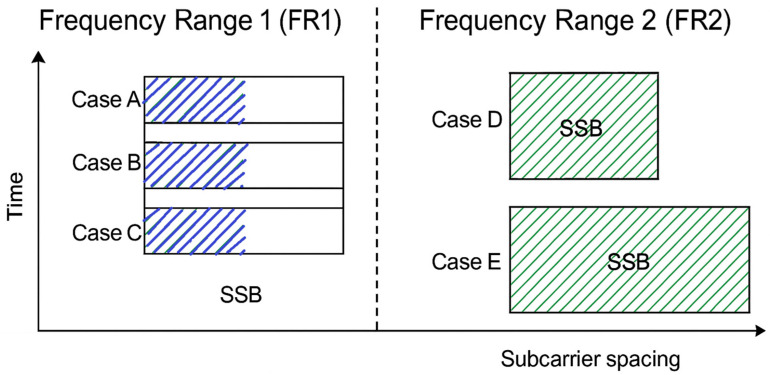
Synchronization signal block patterns.

The gNodeB and UE are the transmitter and receiver ends, respectively, of a 5G NR system. Using an SSB transmitted as a burst in the downlink direction, beam sweeping will establish the best beam pair link. To decide which SSB pattern to use, there are five cases available—Cases A, B, C, D, and E—that are taken into consideration depending on the 5G frequency range that is used, as presented in Figure 5.

Each SSB indicates a certain beam. More SSBs form an SS burst set, which lasts for 5 ms. The burst is then repeated with a period of 20 ms. The first symbol of candidate SSBs is determined based on the subcarrier spacing of the SSB, according to several patterns. If the SCS is not known, the appropriate SSB pattern for the cell depends on the frequency band. The maximum number of synchronization signal blocks in a single burst varies depending on the frequency. The gNodeB regularly transmits a burst of beams in all directions. When the UE is synchronizing with the network, it reads the SSB. The spectrogram characteristics of the generated SSBs under various configurations, ranging from traditional sub-6 GHz frequencies (FR1) to millimeter wave (FR2) scenarios, are examined.

The analysis focuses on how different parameters—such as subcarrier spacing, frequency band, and number of transmitted SSBs—influence the signal’s behavior in the time-frequency domain. Table 3 displays the main parameters adopted for the two use cases analyzed.

**Figure 6:**
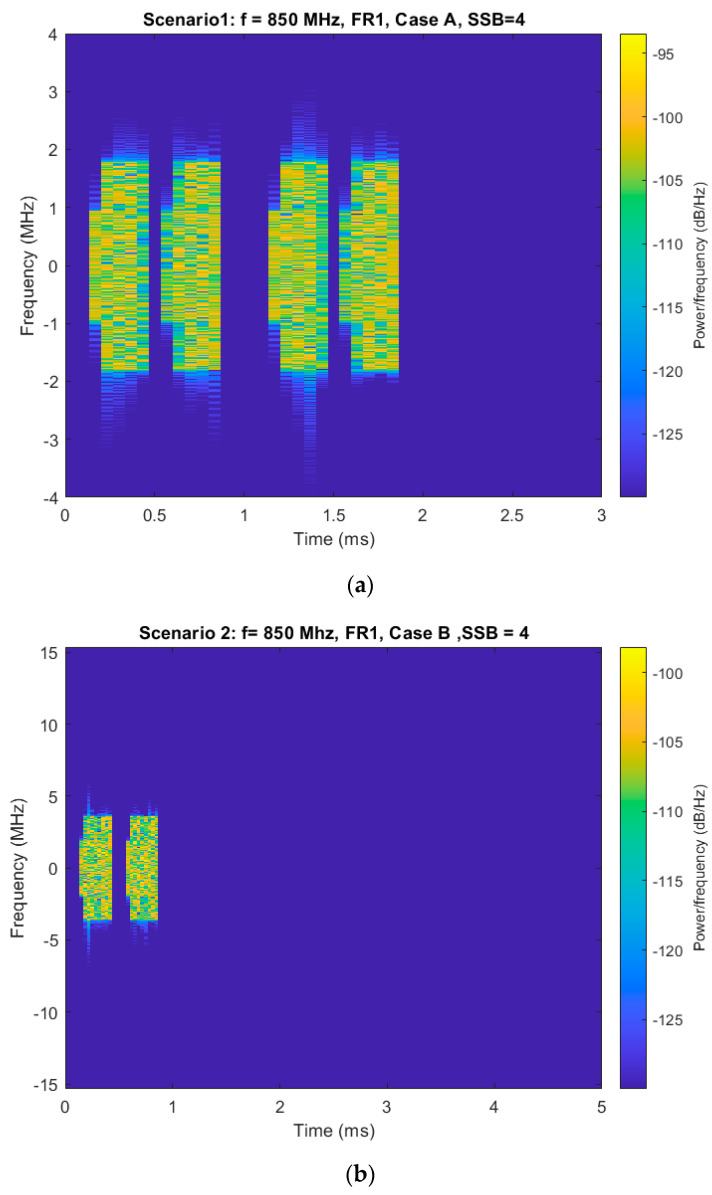

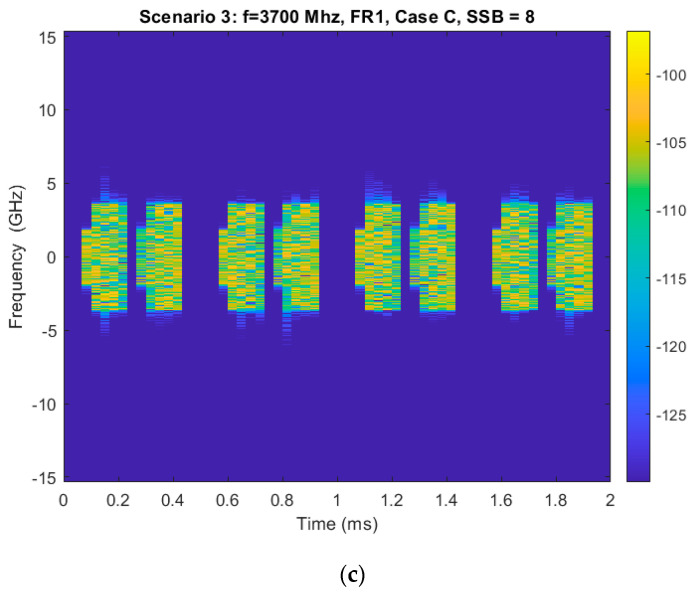
(**a**) Spectrogram of generated SSB—scenario 1. (**b**) Spectrogram of generated SSB—scenario 2. (**c**) Spectrogram of generated SSB—scenario 3.

The first three scenarios emphasize the spectrogram of generated SSBs corresponding to 5G FR 1. Scenario 1 utilizes frequency band 850 MHz with a 15 kHz SCS and 4 transmitted SSBs, which means Case A must be used. The SSB spectrogram appears narrowband with low frequency dispersion, as presented in Figure 6a.

The small number of SSBs results in a few time-domain bursts, while the low SCS ensures minimal frequency occupation, making this configuration appropriate for legacy and low-bandwidth applications.

Scenario 2, also in FR1 at 850 MHz, doubles the subcarrier spacing to 30 kHz (Case B). Although the number of SSBs remains at four, each occupies more frequency space, resulting in a spectrogram that shows slightly wider spectral activity while maintaining similar time-domain sparsity (Figure 6b). This provides a balance between bandwidth efficiency and synchronization precision.

In scenario 3, a significant change is introduced with the carrier frequency increased to 3700 MHz and eight SSBs transmitted under Case C. Operating still in FR1, this scenario demonstrates higher spectral occupancy and denser time-domain activity (Figure 6c). The increase in both SSB count and center frequency yields a more active and broader spectrogram, reflecting enhanced beam coverage for mid-band deployments.

**Figure 7:**
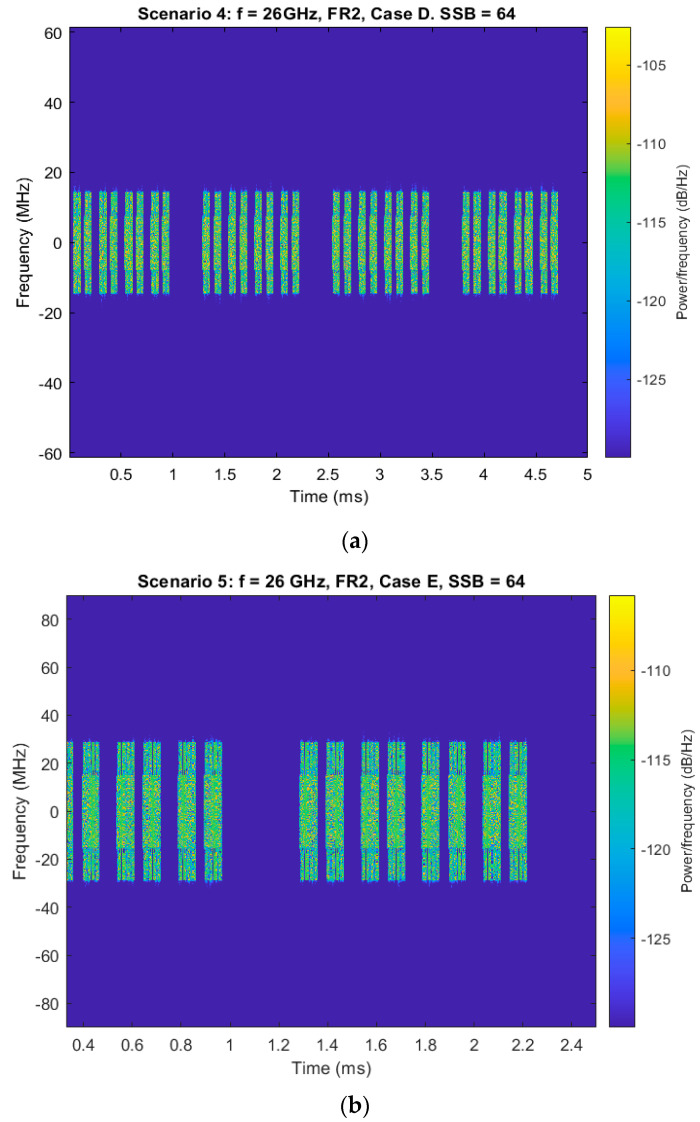
(**a**) Spectrogram of generated SSB—scenario 4. (**b**) Spectrogram of generated SSB—scenario 5.

The transition to scenario 4 highlights a shift to millimeter wave operation, meaning FR2, at 26 GHz with a 120 kHz SCS and 64 transmitted SSBs, resulting in the use for Case D. In this scenario, the spectrogram is significantly denser and wider, with time-frequency bursts closely packed, as seen in Figure 7a. The large number of SSBs and higher SCS boost time-domain density and spectral spread, allowing for fine-grained beam sweeping, which is critical for initial access in highly directed mmWave environments.

Finally, scenario 5 further extends the frequency-domain activity by doubling the SCS to 240 kHz while maintaining 64 SSBs at 26 GHz and using Case E. The result is a spectrogram illustrated by very wide spectral bursts and compact time-domain transmissions, Figure 7b. This configuration maximizes synchronization speed and spectral efficiency, making it ideal for massive beam management and ultra-reliable and low-latency communications in next-generation networks.

Across the five analyzed scenarios, a consistent pattern becomes known: as the subcarrier spacing and the number of transmitted SSBs increase, the spectrogram becomes wider and more densely populated. This evolution facilitates improved synchronization accuracy, accelerates beam sweeping, and enhances the reliability of initial access—capabilities that are particularly crucial in 5G NR deployments. These results underscore the importance of precisely configuring beamforming and synchronization parameters to optimize network performance according to the specific deployment environment and service requirements.

**Figure 8:**
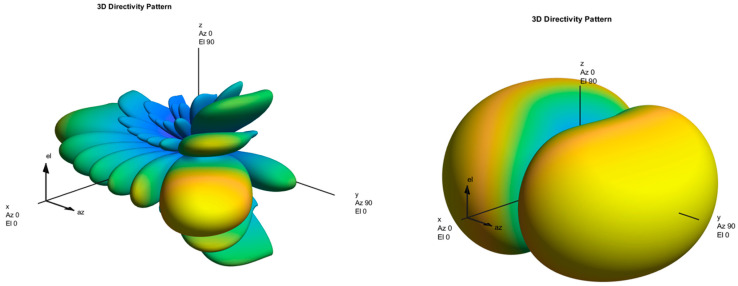
Tx-Rx 3D directivity patterns.

Additionally, the corresponding directivity patterns for both the transmitter and receiver are illustrated in the subsequent Figure 8. It can be observed that multiple beams are transmitted in different directions from the gNodeB to identify the optimal transmission path to the UE. This visual representation highlights the key stages of beam management: beam sweeping and measurement, beam determination, beam reporting, and beam recovery. The varying lobe widths in the figure reflect the process of refining beam directions to achieve precise user targeting, essential for maintaining high-quality links when dealing with high-frequency bands.

The large central lobe from the Tx directivity pattern, which indicates the main beam, represents the direction in which most of the transmitted power is concentrated. This main lobe is critical for delivering the highest possible signal strength to the intended UE, optimizing the SNR, and reducing unnecessary radiation in other directions. The side lobes, while smaller, represent secondary radiation patterns and are an inevitable consequence. These lobes are minimized through proper beamforming weight design to prevent interference and power leakage. The shape and width of the main lobe directly influence beam precision and coverage: a narrower lobe ensures high spatial resolution and focused transmission, whereas a wider lobe, like the one in this figure, provides more coverage, useful for initial access and synchronization where the exact user direction is not yet known. The gradual narrowing of the lobes from the gNodeB to the Rx demonstrates the transition from wide search beams used during sweeping to fine-tuned beams during beam refinement and tracking.

On the other hand, for the Rx directivity pattern, on the right side of Figure 8, a dual-lobe structure is observed. This shows that the RX is configured to be most sensitive where signal strength is expected to be the maximum. The color gradient (from blue to yellow) visually encodes the gain intensity, with yellow regions corresponding to high-gain areas, further validating the Rx’s beamforming focus.

Moreover, the alignment between the Tx and Rx main lobes is crucial—it indicates a successful beam pair, which forms the basis for high-throughput and reliable communication in 5G NR systems.

Table 4 complements the above visual analysis by presenting the RSRP values recorded across the five simulation scenarios, each representing different frequency ranges and beam configurations. The results show a clear relationship between beam directivity and RSRP performance. The scenario in FR1 (e.g., Scenario 1 and 3) report high RSRP due to favorable propagation and wider beams that enable more robust link establishment. In contrast, FR2 scenarios (e.g., Scenario 4 and 5), despite offering higher directivity with 64 SSBs and tighter beams, exhibit lower RSRP due to increased path loss and signal attenuation in the mmWave range. While a favorable relationship between beam directivity and RSRP is theoretically expected, the simulation provides more contextualized, scenario-specific information. Table 4 shows that scenarios with stronger directivity (e.g., Scenarios 1 and 3) have much higher RSRP values (~55.7–55.8 dBm) than those with wider beams or more obstructed routes (e.g., Scenarios 2, 4, and 5: ~25–32 dBm). This demonstrates that beam sharpness is a major factor in increasing received signal intensity under line-of-sight (LOS) situations. These findings highlight the importance of balancing beam sharpness with environmental conditions and emphasize the critical role of both TX and RX beam patterns in optimizing 5G link performance.

## 5. Conclusions

The use of beamforming techniques in 5G systems has the potential to improve future wireless communication networks. A concise description and classification of beamforming techniques were provided. Fifth-generation systems may employ a variety of beamforming techniques, including implicit and explicit beamforming, wideband and narrowband beamforming, as well as fixed and adaptive beamforming. Beamforming using phased array antennas enables precise directional transmission, significantly enhancing SNR, minimizing interference, and improving spectral efficiency. Effective beam management procedures—such as initial beam establishment, beam sweeping and measurement, beam determination, beam reporting, and beam recovery—are critical in maintaining robust connectivity between the UE and gNodeB, especially for dynamic channel conditions. The ability of the network to select the optimal beam pairs ensures high-quality communication, even in challenging environments. Furthermore, the spectrogram analysis of SSBs in several deployment scenarios across FR1 and FR2 exposes the adaptability of 5G’s time-frequency structure. These findings clear up how different configurations affect the temporal and spectral occupancy of synchronization signals, with direct consequences for cell identification, initial access, and energy efficiency.

In conclusion, the synergy between advanced beamforming strategies, intelligent beam management, and adaptive synchronization signal configuration forms the cornerstone of 5G’s ability to deliver high-throughput, low-latency, and highly reliable wireless communication.

## Figures and Tables

**Table 1:** An outlook on beamforming techniques based on different criteria.

Criteria	Beamforming Techniques		
Weight vector application	Fixed Beamforming	Adaptive Beamforming	
Location	Transmit Beamforming	Receive Beamforming	
Signal Bandwidth	Narrowband Beamforming	Wideband Beamforming	
Channel Estimation	Implicit Beamforming	Explicit Beamforming	
Signal Domain	Frequency/Time Domain Beamforming	Space-time Beamforming	
		Analog Beamforming	Digital Beamforming

**Table 2:** In-depth analysis of beamforming techniques.

Beamforming Technique	Methodology
Fixed Beamforming	This technique uses a predefined set of weight vectors to control the radiation pattern of an antenna array. It is suitable in systems where simplicity, low complexity, and fast switching between beams are required, such as in 5G SSB transmission, Wi-Fi access points, radar systems, and some satellite communication systems. A collection of predefined weight vectors dictates the radiation pattern of the antenna elements. Depending on the DOA estimation, the fixed beamforming system selects which weight vector to apply to the signal.
Adaptive Beamforming	It is a smart antenna technique where the antenna array can dynamically adjust its weight vectors and radiation patterns in real time. This dynamic adaptation enables the system to steer the main lobe of the radiation pattern towards a desired signal source while simultaneously minimizing interference from undesired directions (null steering). Adaptive beamforming uses algorithms—such as the least mean squares (LMS), recursive least squares (RLS), or minimum variance distortionless response (MVDR) to constantly update the weights in real time. Using adaptive algorithms at both the transmit and receive ends can increase SNR [14].
Transmit Beamforming	At the transmitter, beamforming can be applied between the signal source and the radiating elements to steer the electromagnetic field in three-dimensional space. Adaptive transmit systems improve energy and spectral efficiency. This is achieved by dynamically optimizing the beam pattern based on real-time channel state information (CSI). These systems focus energy on users with higher precision, reduce power consumption, and reuse frequency bands more effectively [15].
Receive Beamforming	It works by combining the signals received from multiple antenna elements in such a way that constructive interference increases the desired signal while destructive interference suppresses noise and unnecessary signals. This process applies weight vectors to each antenna input—adjusting the phase and amplitude—before summating them. The resulting beam is steered toward the Direction of Arrival of the desired signal, maximizing SNR [14].
Narrowband Beamforming	Beamforming associated with narrowband signals can be achieved through the immediate linear combination of the signals received by the array. Presently, standard wireless technologies are mainly concentrated on narrowband beamforming, which is increasingly becoming a crucial component of the 5G network. The required temporal adjustments in the mapping matrix for narrowband signals are remarkably simple. This is derived from the observation that altering the phase of a narrowband signal is akin to modifying time [11].
Wideband Beamforming	In the case of wideband signals, the phase shifting networks might not be adequate to yield the expected output. The conventional approach for wideband array processing involves the introduction of taps, followed by the determination of their suitable weights for the beamforming procedure. Mm-wave beamforming serves as an excellent example of wideband beamforming, which can be utilized in 5G to achieve extraordinarily high speeds and substantial capacity [16].
Implicit Beamforming	In implicit or open-loop beamforming, the transmitter carries out a channel sounding process that relies on the assumption of channel reciprocity. It dispatches training sequences to adjust the phase-shift variances caused by multipath, with the aim of computing the components of the steering matrix. This method’s merit lies in its lower overhead, and it proves effective in situations where the transmitter might possess superior array processing abilities compared to the receiver [17].
Explicit Beamforming	In explicit or closed-loop beamforming, the receiver gauges the channel, and the data is relayed back to the transmitter. There are three frequently employed modes of explicit beamforming: (a) the uncompressed mode, where the feedback consists of the steering matrix as determined by the receiver, (b) the compressed mode, where the receiver transmits a compressed steering matrix to the transmitter, and the CSI feedback, in which the receiver merely returns the raw channel estimates to the transmitter for the computation of the steering matrix [18].
Frequency Domain Beamforming	This technique implies the application of the Fourier transform to the beamforming equation. Thus, each beam represents a weighted linear combination of the Fourier transform coefficients of the received signals. The number of beams that can be formed is at least equal to or greater than the number of antenna elements, and it is not restricted by the sampling period. Frequency domain implementations are employed to reduce the cost and size of the system, but they demand more processing power.
Time-Domain Beamforming	The inverse Fourier transformation is used to obtain time-domain beamforming. In this type of beamforming, the sensor wave fronts are shifted or delayed, shaping one beam. In this case, the sampling period limits the number of beams that can be formed. This method is adaptable for any array geometry that has a smaller number of antenna elements compared to frequency-domain beamforming [19].

**Table 3:** List of parameters.

Parameters	Scenario 1	Scenario 2	Scenario 3	Scenario 4	Scenario 5
Frequency range	FR1	FR1	FR1	FR2	FR2
Frequency band	850 MHz	850 MHz	3700 MHz	26 GHz	26 GHz
SSB	Case A	Case B	Case C	Case D	Case E
SCS	15 kHz	30 kHz	30 kHz	120 kHz	240 kHz
SSB transmitted	4	4	8	64	64
SNR	50 dB	50 dB	50 dB	50 dB	50 dB
UE Noise Figure	10 dB	10 dB	10 dB	10 dB	10 dB

**Table 4:** RSRP values for the different scenarios.

Parameters	Scenario 1	Scenario 2	Scenario 3	Scenario 4	Scenario 5
RSRP	55.72 dBm	31.34 dBm	55.80 dBm	25.31 dBm	32.08 dBm

## Data Availability

Data sharing not applicable.

## Abbreviations

The following abbreviations are used in this manuscript:

ABP	Auxiliary Beam Pair
BER	Bit Error Rate
BF	Beamforming
BFU	Beamforming Unit
BM	Beam Management
BS	Base Station
CSI	Channel State Information
DMRS	Demodulation Reference Signal
DoA	Direction of Arrival
eMBB	Enhanced Mobile Broadband
FDD	Frequency Division Duplex
FM	Frequency Modulation
FR	Frequency Range
FR1	Frequency Range 1
FR2	Frequency Range 2
GNodeB	Next Generation Node B
HB	Hybrid Beamforming
LMS	Least Mean Square
MIB	Master Information Block
MIMO	Multiple Input Multiple Output
mMIMO	Massive Multiple Input Multiple Output
mmWave	Millimeter-Wave
MVDR	Minimum Variance Distortion Less Response
NR	New Radio
PBCH	Physical Broadcast Channel
PSS	Primary Synchronization Signal
RLS	Recursive Least Squares
Rx	Receiver
SNR	Signal to Noise Ratio
SNoI	Signals Not of Interest
SoI	Signal of Interest
SRS	Sounding Reference Signal
SSB	Synchronization Signal Block
SSS	Secondary synchronization signal
Tx	Transmitter
UE	User Equipment
